# Indigenous Knowledge and the Microbiome—Bridging the Disconnect between Colonized Places, Peoples, and the Unseen Influences That Shape Our Health and Well-Being

**DOI:** 10.1128/msystems.00875-22

**Published:** 2023-01-25

**Authors:** Isaac Warbrick, Deborah Heke, Martin Breed

**Affiliations:** a Taupua Waiora Centre for Māori Health Research–Auckland University of Technology, Auckland, New Zealand; b College of Science and Engineering–Flinders University, Adelaide, Australia; The University of Maine

**Keywords:** environmental microbiome, Māori Health, Indigenous health, Indigenous knowledge, Maori health, planetary health, environmental microbiology

## Abstract

Indigenous Peoples have a rich and long-standing connection with the environments that they descend from—a connection that has informed a deep and multifaceted understanding of the relationship between human well-being and the environment. Through cultural narratives and practices, much of this knowledge has endured despite the ongoing effects that colonization has had on many Indigenous peoples across the world. These narratives and practices, based on observation, experimentation, and practical application over many generations, have the potential to make compelling contributions to our understanding of the environmental microbiome and its relationship to health. Furthermore, the inclusion of Indigenous perspectives regarding the microbiome opens pathways to those who rarely engage with the field and its learnings. Within the scientific community, Indigenous perspectives have not always been acknowledged as valid contributions and are often seen as myth or lacking rigor. Thus, this paper aims to explore an Indigenous perspective of the microbiome as an unseen influence on health and well-being by framing the importance of the natural environment, Indigenous knowledge and leadership, and future research directions that can contribute to this domain. Although the Indigenous perspective in this article reflects the experiences, worldviews, and knowledge of two New Zealand Māori authors, it is hoped that the concepts discussed can relate to Indigenous peoples, and non-Indigenous advocates, globally.

## INTRODUCTION

Tuia ki te rangi, tuia ki te whenua, tuia ki te moana, tuia te here tangata.

Ka rongo te pō, ka rongo te ao.

Though subtle nuances of te reo and whakaaro Māori (Māori language and ways of knowing) are lost in translation to English, this karakia/whakataukī (incantation/proverb) is an invocation to bind together the skies (rangi), the land (whenua), the seas (moana), and people (tangata), those things that are unseen (pō), and those things that are seen (ao), while recognising a balance between male and female. Colonization has driven Indigenous Peoples globally from their ancestral lands, and the environments that sustained them for generations. Despite the “confiscation” and subsequent reduced access to their lands, Indigenous Peoples have maintained a spiritual and emotional connection to mountains, rivers, oceans, forests, and other features of the environment, at times without physically setting foot in those lands. Indigenous Peoples may even share physiological connections with their ancestral environments, as shown through adaptations associated with exposure (1). Indeed, Indigeneity has been defined by a unique connection to land and features of the environment (2). It is therefore no surprise that Indigenous Peoples who are displaced from ancestral lands experience poorer health, education, and other social measures, compared to their non-Indigenous “neighbors” (3, 4). While colonization has had devastating and obvious effects on Indigenous People’s health and wellbeing, here we focus on the less obvious and much less visible outcomes of this forced disconnection on the health of Indigenous Peoples—disconnection from a diverse environmental microbiome.

In addition to suffering from inequities in access to health services, healthy foods, and adequate housing (5–7), it is also likely that Indigenous Peoples have reduced access and exposure to health-promoting microbiota. As a result of urban drift and the impacts of urbanization on the environmental microbiome (8, 9), many Indigenous People now live in cities that are far from their ancestral lands and the diversity of potentially beneficial exposures they possess (10). What’s more, Indigenous Peoples, like many minority groups, are more likely to have reduced access to high quality and/or biodiverse green and blue spaces in urban areas due to their greater representation in lower socioeconomic neighborhoods (11, 12) and the general impacts of urbanization on ecosystem integrity and biodiversity (9). On the other hand, eating more traditional diets is associated with increased diet quality (13) and improved cardiovascular health (14) among Indigenous Peoples and a traditional Indigenous lifestyle has been associated with more diverse and abundant commensal microbiota (15). Arguably, many “[p]eople have lost their way as a human species as they have forgotten that they are Nature. Regaining this relationship with ourselves and Mother Earth is crucial for the wellbeing of our planetary home” and indeed human wellbeing (16).

Indigenous narratives may not refer directly to the microbiome, but there are many references to an unseen connection between people and the environment. For example, Indigenous Peoples may talk of the environment “speaking to us,” and similar language is a part of other traditions, including within the many major religious texts. Drawing upon messages from her Elders, N. Redvers (17) writes

When mother trees are dying, they send messages of wisdom on to the next generation of seedlings. She sends these messages through a vast internet-like fungal network in the soil to her neighboring seedlings. This increases the resistance of these seedlings to future stresses, which in essence means that trees talk.

Although Indigenous perspectives, such as these, have been looked upon as mystical or mythical compared with western science, researchers of the microbiome also use language such as “cross talk between the microbiota and the surrounding host environment” (17).

This paper aims to (i) reframe the connection between environmental microbiomes, human microbiomes, and health within an Indigenous paradigm; (ii) emphasize the values of Indigenous knowledge, leadership, and participation in microbiome research; and (iii) discuss important considerations for future research that has the potential to make substantial progress in this domain. Isaac Warbrick is of Ngāti Te Ata, Te Arawa, and Ngā Puhi descent (Māori iwi [tribes] of New Zealand’s North Island), and Deborah Heke is of Ngā Puhi and Te Arawa descent. Both Warbrick and Heke are health researchers with a background in exercise physiology and the application of Māori knowledge to improve health and wellbeing, at Auckland University of Technology. Martin Breed is a scholar of restoration ecology, ecosystem health, and genomics at Flinders University, Australia. Although the Indigenous perspective in this article reflects the experiences, worldviews, and knowledge of two NZ Māori authors, it is hoped that the concepts discussed can relate to Indigenous Peoples and non-Indigenous advocates, globally.

## THE UNSEEN OF INDIGENOUS HEALTH IS “IN THE ENVIRONMENT”

In New Zealand, Māori are disproportionately affected by non-communicable diseases (NCDs) such as cancers, diabetes, metabolic illness, and heart disease, like many Indigenous Peoples globally (18). Interestingly, recent research has shown that many NCDs are associated with and influenced by the microbiome (19–22). For example, dysbiosis of gut microbiota was present in stroke and transient ischemic attack patients, compared with controls (23), and reduced diversity of the gut microbiome was associated with obesity and insulin resistance (24).

Alternatively, engagement with natural environments is associated with many health benefits. For example, time spent in and around green spaces (a term often relating to parks and forested areas within urban centers) is associated with improved management of stress, increased physical activity levels, and a lower risk of chronic illness (25). Urban nature interventions have potential in contributing to such health benefits with relatively low cost (26), especially considering the global population living in cities is projected to reach close to 70% by 2050 (27). Being outdoors also increases exposure to (28) and transfer of environmental microbiomes onto the skin (29), via the aerobiome (30), the respiratory tract (29) and into the gut (19). For example, a placebo-controlled double-blinded study in Finland showed that the intervention group that engaged with microbially rich soil brought into playground sand experienced colonization of these microbiota to the skin with subsequent promotion of immunomodulation (interleukin-10 and T cell frequencies) (19).

Indigenous Peoples are particularly vulnerable to climate change, and the climate crisis will likely compound health inequities experienced by Indigenous People (31, 32). Contemporary climate change is also directly impacting the ability of Indigenous People to interact with ancestral lands. For example, Maldonado et al. (33) highlighted the case of the Isle de Jean Charles in Louisiana, where intense coastal erosion has reduced the island that has been a home and refuge to Grand Caillou/Dulac Band of Biloxi-Chitimacha-Choctaw since they were forced to relocate there by early settlers. Accordingly, microbiota also respond directly to changes in climate but also indirectly via the degrading effect of climate change on ecosystems (34). Therefore, as more Indigenous People become urban-based or disconnected from ancestral lands, their interaction with the natural environment becomes less frequent. What’s more, the contemporary environments that many of us now inhabit are vastly different from those of our ancestors. Contemporary diet and lifestyles are also now more sterilized, polluted, and/or microbially impoverished than ever before (35), and our ancestors were likely exposed to a diversity of environmental microbes (36) via interactions with nature and associated dietary practices (37).

Inherent in the ancestral interactions with nature came the creation of kōrero tuku iho (traditional teachings and wisdom that is passed down through subsequent generations). These traditional teachings contained *and still carry* meaningful sources of mātauranga (knowledge) or messages about the structures of nature and how to successfully navigate them. Indigenous narratives are replete with examples connecting the health of individuals and communities with the natural environment. For example, Inyang refers to a proverb from the Ibibio of Kenya—“ke owo aba nte nkankuk omo”—which suggests that a person’s life is “replicated” in their environment (38). Luther Standing Bear of the Sicangu and Oglala Lakota once noted that “we are of the soil and the soil is of us” (39). Whakataukī (Māori proverbs) often refer to the environment as the source of wellbeing. Water, for example, is portrayed as the life giver of all things in the whakataukī (proverb): “Ko te wai te ora o ngā mea katoa,” or the domain of an unseen lifeforce hidden within the ocean in the whakatauākī: “Ko te mauri, he mea huna ki te moana” (a well-known saying from revered ancestor and ocean navigator Nukutāwhiti). In many cases, the unseen were understood as living entities significant to health and healing. In other cases, features of the environment are personified or given as a metaphor to guide behavior. The reference to the wind as an atua (personified form of the environment or deity) is demonstrated in the instructive whakataukī: Hokia ki ō maunga kia purea ai koe e ngā hau o Tāwhirimātea. This saying encourages people to return to their ancestral mountain/s, where the winds provide replenishment.

Indigenous epistemologies and frames of knowing repeatedly recognize the influence of unseen (or microscopic) forces upon health. Studies of the microbiome can be viewed similarly, as a way of coming to understand those unseen entities that are essential to good health. Some non-Indigenous scientists may be uncomfortable with the “spiritual” connotations associated with Indigenous knowledge systems of the unseen, but for Indigenous peoples, the spiritual and physical worlds are intertwined so that they cannot be fully understood separately (40). Because of this, Indigenous ways of understanding the unseen could provide a culturally relevant framework for the study of the microbiome.

## LEADING INDIGENOUS MICROBIOME RESEARCH

Much of what has been brought to light through the rapid development of micro and molecular technologies (e.g., genomic sequencing technologies), has tended to be interpreted, understood, and presented from non-Indigenous perspectives. Historically, Indigenous People have had little involvement in these fields (41). For the few that are involved, apprehension in applying their Indigenous world view in scientific settings is likely a result of the historical marginalization or undervaluing of Indigenous perspectives as myth or unscientific.

Thankfully there is growing recognition from some academic and publicly funded institutions of the reciprocal advantages of collaboration with Indigenous Peoples (42–44), through the acknowledgment of their deep-rooted cultural ties to land, and the immense value of Indigenous knowledge in areas such as ecology and conservation. This knowledge and its generational connection to sustaining ecosystems is critical to combatting a range of contemporary environmental challenges (45), including access to safe and adequate exposures of health-promoting environmental microbiomes.

The underrepresentation of Indigenous scientists, including Māori in New Zealand, especially within the publicly funded science sector is concerning. The latest available data show Māori comprising between 0% and 8% of the scientific workforce across different institutions (46). Earlier data suggested that as little as 1.7% of scientists identified as Māori in 2008. Data compiled from the U.S. census suggest that microbiologists from American Indian or Native Alaskan descent represent approximately 0.3% of the workforce, while the vast majority are white (~73.8%) (47). There is a paucity of available data on the ethnic representation or diversity of scientists in these organizations, but the lack of diverse representation of Māori and other Indigenous Peoples is also representative of the lack of diverse perspectives in these fields, including microbiome science.

Underrepresentation notwithstanding, there are growing numbers of Indigenous scientists who are conducting microbial research through a uniquely Indigenous lens and/or directed by Indigenous knowledge (48, 49). Aviaja Hauptmann, a microbiologist with Inuit heritage, born in Nuuk, Greenland, now leads research on the Greenland Diet Revolution Project, centering on the fermented and animal-sourced Indigenous diet of the Inuit (50, 51). Chris Pairama and Te Rangitākuku Kaihoro (from Ngāti Whatua and Te Arawa/Ngāti Tūwharetoa, respectively—tribes of New Zealand’s North Island) are leading efforts to incorporate mātauranga (Māori knowledge) into native plant and anti-pathogen identification, as a way to enact kaitiakitanga (environmental stewardship) and combat kauri dieback (Phytophthora agathidicida) (52). Hawaiian scientist Cliff Kapono is using Hawaiian knowledge to inform his approach to genetic and microbiome sequencing. Using the millennia-old practice of He’e nalu (surfing), Kapono’s research in the Surfer Biome Project demonstrates the molecular connection between humans and their environments (53). C. Kapono (53) discusses where traditional Hawaiian knowledge overlaps with western, such that skin microbiome sample variations can be related to the Hawaiian saying *nana I ka ili*, in which the differences and similarities that people have is said to be observed through the skin.

The authors of this article are currently leading a 3-year study in New Zealand exploring environmental observations and knowledge stored in Māori and Pacific narratives and stories, and the use of these narratives in restoring local ecosystems and strengthening the “connection” of Indigenous Peoples to the natural environment. The research team consists of Indigenous elders and experts in traditional knowledge, Indigenous and non-Indigenous researchers from various fields (Indigenous Health and development, microbiology, epidemiology, environmental science, language, and culture), and community experts and leaders, including local Indigenous youth researchers. Though still in preliminary stages of the research process, this study will include a culture-based, community-designed “intervention” to explore the impact of reconnecting with the environment on the microbiome of individual Māori participants. This study also seeks to better understand Indigenous perspectives of and ways of talking about the microbiome, and to build Indigenous research capacity in the study of the environmental microbiome and health.

Beyond the trailblazing work of these and many other Indigenous scientists, and the importance of growing capacity, there is a suggestion, at least in a New Zealand context, that what is required is a “substantive commitment to community partnerships and bicultural research” (45). Not only is there a need for more Indigenous scientists, especially in leadership positions, but there also needs to be a stronger emphasis on the significant role that Indigenous knowledge can have in addressing the colonial history of science. This would ensure that the future workforce is well-equipped with a better understanding of the validity and practice of Indigenous scientific frameworks, free from confinements to a particular discipline, as may have been the case historically (41, 45). Ultimately, the close connection and intimate understanding of environmental connections with human health and wellbeing that is reflective of Indigenous worldviews means it would be unwise to ignore the potential contributions they may have in microbiome science.

## PAST, PRESENT, AND FUTURE RESEARCH CONSIDERATIONS

In a recent article that proposed 20 questions of fundamental importance to achieving equitable exposure to beneficial microbiota, one particular question posed was: “How do research institutions learn to better engage with Indigenous and other marginalized communities in microbiome research?” (42). Here, we outline a few considerations in response to this question.

The ability for Indigenous scientists and researchers to articulate, explain, and understand scientific phenomena through their own cultural lens is an important pathway for Indigenous and non-Indigenous success in any field (54). In addition, the success of Indigenous scientists and researchers creates pathways for people from their own culture to relate and aspire to those roles themselves. Instead of assuming that Indigenous approaches weaken the validity of robust science (55), we argue that Indigenous ontologies, epistemologies, and methodologies provide an increased depth to understand the environment-microbiome-health nexus. Indigenous Peoples have long been able to explain the complexities of natural phenomena through stories and narratives where features of the environment are personified or codified. These uniquely Indigenous mechanisms of understanding and explaining the natural world, including the microbiome and its connection to health and the wider ecosystem—mechanisms underpinned by observation, experimentation, and practical application—have enabled Indigenous Peoples to survive and thrive for generations.

Fad foods and health products made the microbiome popular among healthier and wealthier populations (56), but the benefits of the microbiome and its role as a determinant of health, and in mediating the connection between health and environment, is likely less understood (and utilized) among Indigenous People. It is possible that an Indigenous framing of these growing areas of health could increase the appetite and interest among Indigenous communities, while providing a deeper understanding and motivation to care for the diversity of ecosystems that sustain us. The Māori karakia/poroporoaki (an incantation of sorts) that we began with “Tuia ki te rangi, tuia ki te moana, tuia ki te whenua, tuia te here tangata” is a reminder and acknowledgment of the weaving together of that which is above (rangi), the land (whenua), the oceans (moana), and people (tangata). In this context, the vast microbiome currently measured in the air, water, soil, and within people, plants, and animals, represents one of many physical threads that are woven together with spiritual, cultural, and social threads, to bind us to the environment—a structure consistent with the environment-microbiome-health nexus.

There are also ethical and cultural challenges unique to microbiology that require Indigenous perspectives and leadership (49). Treatment or experimental procedures, such as fecal transplant, may have significant “health” benefits (57), but acceptance and uptake of such procedures among Indigenous Peoples will likely be low without significant Indigenous input. In Māori cultural practice there already exist concepts that govern societal, practical, and hygienic practices. The concept of tapu (state of restriction), for example, provides a uniquely Indigenous way of thinking about cross contamination, and restrictions relating to human waste and food (58). Māori have also developed contemporary ethical guidelines based on cultural values (tikanga), as in Te Ara Tika, a set of guidelines for conducting ethical research with Māori (59); and Te Mata Ira, Guidelines for Genomic Research with Māori, developed to ensure that Māori perspectives and values are reflected in genetic/genomic research (60).

Inequity in the determinants of health are complex, and multipronged approaches are required to achieve more equitable health outcomes for Indigenous Peoples. Nevertheless, based on the close relationship between the microbiome and health, pursuing a better understanding of the microbiome and its role in Indigenous health could contribute toward more equitable Indigenous health outcomes. What’s more, developing strategies that utilize a scientific understanding of the microbiome alongside Indigenous knowledge are likely to produce more meaningful health initiatives with better uptake from Indigenous Peoples and communities.

## CONCLUSION

The study of the microbiome, its connection to all aspects of health and wellbeing, and its role in connecting ourselves with the environment is an exciting field to be involved in. But many who would benefit most from advances in understanding this connection are still sidelined and their perspectives rarely heard or acknowledged as having value in this space. On the other hand, an entire community of researchers and scientists may be missing out on holistic and uniquely Indigenous perspectives, which enrich the obtaining and applying of scientific learning about the microbiome. N. Redvers (17) quotes Chief Dan George as saying, “Allow me to learn the ways of your book knowledge so that I may combine it with my natural knowledge and lead the way.” This combination of traditional knowledge and values, combined with modern scientific procedures and tools, will broaden our perspectives of the microbiome and strengthen the application of our learnings among those who are often marginalized or disenfranchised with scientific enquiry. Sometimes that may mean that it is those from the community who lead the way while scientists or researchers contribute expertise in a supporting role. Ultimately, increasing the cross talk of Indigenous and non-Indigenous scientists and knowledge-holders will ensure that the study of the microbiome and its role in improving health will have further reaching and more equitable effects.
